# Combination of prolonged water fasting and GLP-1 for refractory morbid obesity: Case report

**DOI:** 10.1038/s41430-026-01749-8

**Published:** 2026-04-17

**Authors:** Tiago Palmisano, Paige Roeschenthaler, Heather Passerini, Thomas Shin, David Guarraia

**Affiliations:** 1 Department of Medicine, University of Virginia, Charlottesville, VA USA; 2 Department of Kinesiology, University of Virginia, Charlottesville, VA USA; 3 Department of Surgery, University of Virginia, Charlottesville, VA USA; 4 Department of Cardiovascular Medicine, University of Virginia, Charlottesville, VA USA

**Keywords:** Obesity, Weight management, Nutrition therapy, Nutrition, Bariatric surgery

## Abstract

**Introduction:**

Water fasting involves completely abstaining from all caloric intake. Multiple studies have demonstrated that water fasting for up to 21 days is well-tolerated, but none focused on patients with severe obesity (body mass index ≥ 40) or utilized treatment with glucagon-like peptide-1 receptor agonists (GLP-1).

**Methods:**

The patient was a 44-year-old male with severe morbid obesity, heart failure, oxygen-dependent obesity hypoventilation syndrome, uncontrolled hypertension, anemia, and depression. He underwent a 27 day inpatient fast with weekly tirzepatide, including 21 day water fast with structured refeeding.

**Results:**

The patient lost 125 pounds during the fast. He regained independent ambulation and chronic oxygen supplementation was discontinued. Chronic anemia resolved and depression improved as measured by Patient Health Questionnaire. Systolic blood pressure improved despite discontinuation of his antihypertensives. His chronic leg wound resolved without antibiotics. Left ventricular mass decreased and fasting insulin normalized. Appetite remained tolerable, and the only complication was mild transaminitis.

**Conclusion:**

This case suggests prolonged medically-supervised water fasting with GLP-1 use is an effective short-term weight reduction strategy that allows for bridging to bariatric surgery with improvement in multiple chronic diseases. As the fast ended for surgery rather than intolerance, research into longer water fasts could be considered.

## Introduction

Water fasting is defined as a period of time in which one completely abstains from all caloric intake. Multiple studies have demonstrated that water fasting for up to 21 days is well-tolerated [1, 2], although large studies on time frames longer than this have been limited and, to our knowledge, not incorporated the use of glucagon-like peptide-1 receptor agonists (GLP-1) for appetite suppression. In addition to the beneficial effects on weight loss, prolonged water fasting has been shown to alter the gut microbiome [3], improve blood pressure [4, 5], and lower inflammation markers [6]. A recent review of the literature also showed that prolonged water fasting lowers HbA1c and insulin resistance in normoglycemic patients, but not in those with diabetes [7]. There is also biochemical rationale for a link between fasting and hemoglobin levels [8], and an observational study of religious intermittent fasting demonstrated an increase in hemoglobin [9]. However, fasting also predictably induces compensatory physiologic responses, such as increased appetite signaling and reduced energy expenditure, which may undermine long-term durability. Therefore, the outcomes of extended fasting, particularly in patients with severe obesity, warrant further investigation.

Here we present the case of a 44 year old male with severe premortem obesity (BMI 94; 740 pounds) who underwent a 26 day inpatient medically supervised fast, including 21 days of water-only intake, prior to elective robotic-assisted laparoscopic sleeve gastrectomy. Although some prior research included patients with obesity, the case presented here is by far the most severe case of obesity treated with a documented water fast. To our knowledge, this represents the most severe case of obesity treated with a documented prolonged water fast, and the longest medically planned fast for severe obesity since 1973 [10]. Unlike prior reports of fasting beyond 21 days in non-obese individuals [11, 12], this case demonstrates substantial weight loss (125 pounds) accompanied by improvements in depression, anemia, hypoxia/hypercapnia, functional status, and left ventricular (LV) mass. This latter finding is in accordance with prior research that linked intermittent fasting to both reduced LV mass in mice [13] and to improved LV ejection fraction in patients after ST-segment elevation myocardial infarction [14]. Overall, this case suggests a role for prolonged fasting in combination with a GLP-1 as a bridge to bariatric surgery in carefully selected, highly-motivated patients.

### Case presentation

SL was a 44 year old Caucasian male with a past medical history of severe morbid obesity (BMI 99), heart failure with preserved ejection fraction, oxygen-dependent obesity hypoventilation syndrome (OHS), chronic non-healing leg wounds, anemia, uncontrolled hypertension on 2 anti-hypertensive agents, limited mobility, and major depressive disorder who presented to an academic medical center for treatment of acute on chronic diastolic heart failure due to progressive dyspnea. He had no contraindications to fasting and was motivated to pursue aggressive dietary changes while on his outpatient dose of tirzepatide (7.5 mg weekly). He subsequently received diuretics to treat his acute exacerbation of heart failure with preserved ejection fraction along with medically-supervised fasting to qualify for and proceed to elective, life-saving bariatric surgery.

According to SL, he had completed multiple dietary and exercise programs throughout his adult life without sustained benefit. In the year prior to admission SL participated in two courses of outpatient physical therapy, a set of six visits with a registered dietician for nutritional counseling, and outpatient meetings with the behavioral medicine team. Through this process, his weight decreased from a maximum of 776 pounds (351.9 kg, BMI 99.4) to 745 pounds (337.9 kg, BMI 95.6). His weight then plateaued despite initiation and subsequent escalation of tirzepatide (one month of 2.5 mg per week, followed by one month of 5 mg per week, followed by one month of 7.5 mg per week). At the time of admission he weighed 740 pounds (335.6 kg, BMI 95.1), which had precluded the patient from being a surgical candidate due to concern that the operating-room table would not support his weight, in addition to his severe medical problems listed above. Therefore, the patient agreed to undergo a prolonged water fast in an attempt to optimize his candidacy for bariatric surgery.

**Fig. 1:**
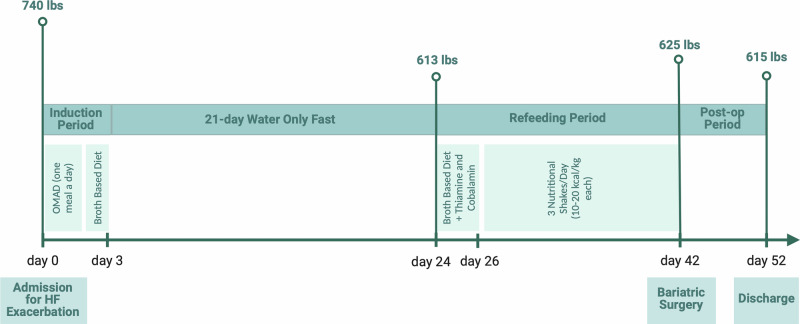
Fasting protocol timeline. The fasting protocol included an induction period consisting of one day of OMAD (one-meal-a-day) and one day of broth-based diet, followed by 21 days of water only. This was followed by a refeeding period consisting of two days of broth-based diet with vitamin supplementation, which then progressed to nutritional shakes until bariatric surgery.

After a three-day induction phase (consisting of low-carbohydrate meals and broth), SL completed 21 consecutive days of water-only intake under close inpatient monitoring. Electrolyte levels and kidney/liver function tests were assessed daily, with appropriate supplementation of sodium, potassium, magnesium, and calcium in addition to administration of a daily multivitamin. Tirzepatide and diuresis were continued, while antihypertensives and statin therapy were held. Hunger was generally mild to moderate (3–6/10), with transient increases when exposed to food stimuli. The only complication was mild transaminitis, attributed in part to acetaminophen use for back pain and headache, which resolved after discontinuation. After 24 days, a refeeding protocol was initiated which consisted of two days of broth with thiamine and cobalamin supplementation, followed by nutritional shakes (10–20 kcal/kg) until surgery. See Fig. 1 for a schematic of the fasting protocol.

**Fig. 2:**
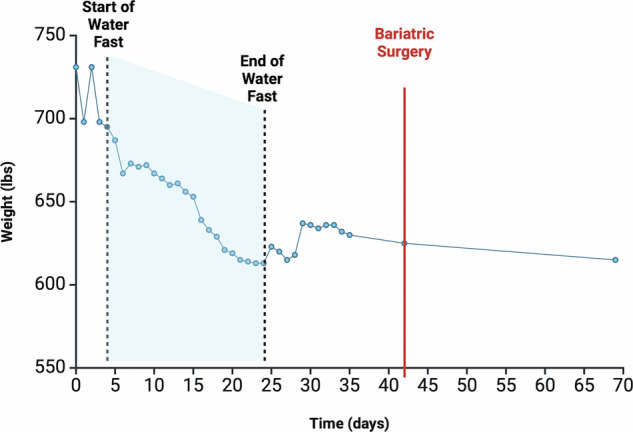
Weight trend during fast. The patient’s weight down-trended in a linear fashion over the course of the fasting, resulting in net 125 pounds of weight loss. His weight increased slightly during the refeeding period, but he returned to his post-fast weight at follow-up after his bariatric surgery.

**Fig. 3:**
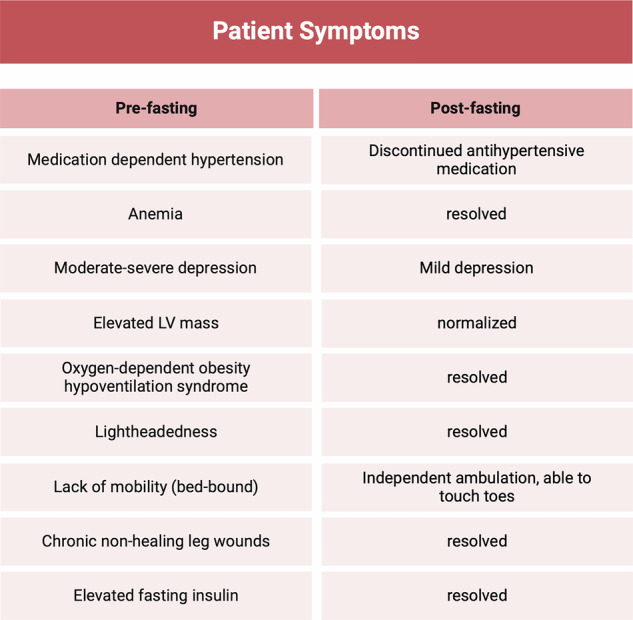
Patient symptoms before and after fasting. The patient had improvement or resolution of multiple chronic medical conditions over the course of his fast including hypertension, anemia, depression, OHS, lightheadedness, poor mobility, and leg wounds. Clinical markers such as LV mass and fasting insulin levels also improved.

By the end of the fast, SL lost 125 pounds (56.7 kg) and had a BMI of 79 (Fig. 2). Despite being bed-bound prior to admission, by the end of his fast he regained ambulation (METS level 4) and was able to touch his toes. His chronic hypoxia/hypercapnia (secondary to OHS) resolved and his home oxygen supplementation was discontinued. His chronic anemia resolved without any iron or blood products, with a hemoglobin increase from 11.7 to 14.7. His chronic depression also improved, with a decrease in his Patient Health Questionnaire (PHQ-9) from 16 to 9. His average systolic blood pressure improved from ~170 to ~140 despite discontinuation of his antihypertensive regimen, and his chronic non-healing leg wound resolved without antibiotics. LV mass as measured by transthoracic echocardiogram decreased from 249.6 grams (three months prior to his fast) to 225.2 grams. His fasting insulin level decreased from 25.6 to 14.9, representing improved insulin resistance (Fig. 3). His troponin and brain natriuretic peptide (BNP) levels were unremarkable. Serum beta-hydroxybutyrate level increased steadily during his admission, representing adherence with the protocol. As expected with fasting, uric acid also increased (peak of 16) and down-trended after refeeding, and the patient did not develop any signs of gout.

As the patient entered the third week of his water fast, he was re-evaluated by the bariatric surgery team and was determined to now be an appropriate candidate for sleeve gastrectomy after his significant weight reduction. Therefore the patient elected to end his fast, and after the predefined refeeding period he underwent successful robotic-assisted laparoscopic sleeve gastrectomy. His post-op course was complicated by one episode of fever and diffuse erythematous rash, diagnosed as drug-reaction to prophylactic perioperative vancomycin, and the patient did not have any further complications. He was discharged on hospital day 52, and at telemedicine follow-up SL was adhering to a standard post-bariatric surgery diet and had continued to lose weight (Fig. 2). When asked for his perspective on the fast, SL described it as “something that needed to be done” and stated he was satisfied with the results. He denied any negative experiences or suggestions for improvement of the protocol, and also denied any lingering adverse effects two months after completion of the fast.

## Discussion

This is the case of a 44 year old man with extreme, refractory obesity who successfully underwent a 21 day water fast in combination with GLP-1 treatment prior to bariatric surgery. In a prior large observation study by Wilhelmi de Toledo et al. the average BMI in the 20 day fasting group was 33.6 (class 1 obesity), while a smaller study by Dai et al. completely excluded patients with obesity [1, 2]. Therefore, this report represents the first documented prolonged water fast for a patient with morbid obesity (BMI ≥ 40) since 1973, when Angus Barbieri lost 276 pounds (82 kg) over 382 days [10]. Minor complications during SL’s fast included transaminitis (which improved after the fast was completed) and headaches. These issues were mild and did not warrant discontinuation of the fast. This case demonstrates that prolonged water fasting with concurrent GLP-1 use is likely both effective and safe for the purpose of short-term weight reduction as a bridge to bariatric surgery. However, additional research is necessary to ensure these findings are reproducible, and the durability of fasting-induced weight loss without ongoing therapeutic support remains uncertain.

SL’s case is also in accordance with prior research showing that prolonged fasting can improve blood pressure [4, 5] and insulin resistance in patients without diabetes [7], suggesting that fasting can transiently improve cardiometabolic parameters. The spontaneous resolution of the patient’s chronic anemia was unexpected, but does fit with research by Luo et al. showing a positive relationship between ferroportin 1 expression (which increases hemoglobin) and ghrelin (which is upregulated during fasting) [8]. His reduction in LV mass carries clinical significance as increased LV mass has been associated with a heightened risk for heart failure hospitalizations and cardiovascular death in heart failure with preserved ejection fraction [15]. Of note, a recent study by Packer et al. supports the use of GLP-1s for the treatment of heart failure with preserved ejection fraction, perhaps through a similar mechanism [16]. However, it is worth noting these improvements may reflect the physiologic consequences of rapid weight loss rather than reversal of underlying disease mechanisms, and whether such changes would persist without definitive obesity treatment is unknown.

In addition to the objective improvements in his biomarkers, SL’s quality-of-life benefits should not be overlooked. His PHQ-9, a marker for depression severity, decreased dramatically over the course of the fast. This was likely driven in large part by the patient’s rapid improvement in functional status and autonomy, as he regained the ability to walk and his need for supplemental oxygen resolved completely. Of course, these results were dependent on SL’s ability to adhere to the strict nature of a water fast. The fact that SL’s hunger remained mild to moderate through most of his fast, despite multiple weeks without caloric intake, was likely assisted by the use of the GLP-1 tirzepatide during the fast. Although tirzepatide had previously failed to stop the progression of SL’s obesity, his case suggests that GLP-1 therapy may help mitigate hunger during periods of enforced caloric restriction, potentially improving tolerability of short-term fasting used for procedural optimization. The lack of any of the traditional adverse effects associated with GLP-1s such as nausea/vomiting or pancreatitis (amylase and lipase levels were monitored) also support the safety of its use during fasting.

Of course, prolonged water fasting is not a suitable treatment option for all patients with obesity. There is likely a subset of patients, especially those such as SL, whose genome predisposes them to the development of severe refractory obesity. The understanding that obesity is a polygenic disease is well-established, with leptin (LEP), leptin receptor (LEPR), melanocortin 4 receptor (MC4R), and proopiomelanocortin (POMC) being prominent contributors. Monogenic obesity was excluded in SL prior to the initiation of the fast. Future research assessing the effect of prolonged fasting on obesity-related gene expression may help elucidate which patients would benefit from prolonged fasting. Furthermore, although inpatient monitoring was necessary in SL’s case given his acute on chronic heart failure necessitating diuretic adjustment, other patients may tolerate an outpatient protocol. It is also worth noting that SL did not end his fast due to intolerance or complication, but rather because his weight loss made him a candidate for bariatric surgery. Therefore, future research into the tolerability and effectiveness of water fasting longer than 21 days could be considered.

## Conclusion

This case report demonstrates that the combination of prolonged water fasting and a GLP-1 receptor agonist is a safe and effective method for short-term substantial weight loss in patients with severe obesity for the purpose of pre-procedural optimization prior to bariatric surgery, and may result in the improvement of multiple chronic diseases including hypertension, insulin resistance, anemia, depression, and diastolic heart failure.
